# The role of culturally appropriate interpersonal communication strategies to reduce hepatitis B and liver cancer disparities

**DOI:** 10.3389/fpubh.2024.1377096

**Published:** 2024-08-09

**Authors:** Beatrice Zovich, Suzanne J. Block, Fiona Borondy-Jenkins, Kate Moraras, Thomas Chen, Rukayat Adedokun, Dung Hua, Chari Cohen

**Affiliations:** ^1^Hepatitis B Foundation, Doylestown, PA, United States; ^2^Department of Health, Behavior and Society, Johns Hopkins Bloomberg School of Public Health, Baltimore, MD, United States; ^3^Department of Internal Medicine, The Mount Sinai Hospital, New York, NY, United States; ^4^Vital Access Care Foundation, Fountain Valley, CA, United States

**Keywords:** hepatitis B, liver cancer, disparities, hepatocellular carcinoma, immigrants, awareness, health education, communication campaign

## Abstract

**Introduction:**

Asian and Pacific Islander (API), African, and Caribbean immigrant groups in the U.S. are disproportionately impacted by chronic hepatitis B and hepatocellular carcinoma (primary liver cancer). Creating educational communication campaigns about hepatitis B and liver cancer for these communities is necessary to increase disease-related awareness and prompt health-promoting behaviors. Identifying interpersonal communication (IPC) preferences within diverse communities for integration into an educational campaign that emphasizes the link between hepatitis B and liver cancer can ultimately promote uptake of screening, vaccination and linkage to appropriate care.

**Methods:**

Fifteen focus groups and two key informant interviews were conducted with participants from Micronesian, Chinese, Hmong, Nigerian, Ghanaian, Vietnamese, Korean, Somali, Ethiopian, Filipino, Haitian, and Francophone West African communities. Data were analyzed using thematic coding and analysis.

**Results:**

Findings demonstrate that all communities preferred that materials be offered in both English and native languages and emphasized that campaigns highlight the connection between hepatitis B and liver cancer. Educational sessions should take place in settings where communities feel safe, including community-based organizations, religious establishments, and healthcare offices, and should be facilitated by trusted messengers, including patient navigators, doctors and faith leaders. Presenting accurate information and dispelling myths and misconceptions around hepatitis B, liver cancer, and their connection were the biggest needs identified across all focus groups.

**Discussion:**

This study provides insight into community-specific preferences for learning about hepatitis B and liver cancer through IPC methods. The findings from this study can be used to design multi-platform, culturally and linguistically appropriate health education campaigns to facilitate improved diagnosis, prevention, and management of hepatitis B and liver cancer among heavily impacted communities in the U.S.

## 1 Introduction

Asian and Pacific Islander (API), African, and Caribbean immigrant groups in the U.S. remain disproportionately impacted by chronic hepatitis B and hepatocellular carcinoma, or liver cancer (1–4). And while these groups shoulder the majority of the burden of chronic hepatitis B in the U.S., most remain unaware of their infection. Rates of hepatitis B screening and vaccination remain low, as do rates of appropriate linkage to sustainable medical care, including treatment uptake to prevent disease progression, and screening for liver cancer, of which hepatitis B virus is a leading cause worldwide (5–8). Tailored health messaging campaigns can play an important role in facilitating hepatitis B- and liver cancer-focused health behaviors.

Interpersonal communication (IPC) strategies are fundamental public health tools for the dissemination of health information. IPC theories focus on understanding how relational processes shape health behaviors (9). For instance, when there is an established relationship built on trust between people, an individual receiving a message encouraging them to engage in a specific health behavior may be more likely to do so. Consequently, messengers and communication channels are important in supporting or inhibiting health behavior change and are likely diverse and distinct within differing communities. IPC can be verbal or nonverbal and includes any type of direct exchange between people (9). Culturally sensitive interpersonal communication strategies, including employing the audiences’ preferred language, identifying and respecting influential psychosocial and cultural factors, and engaging with communities with cultural humility, are all ways public health practitioners can overcome challenges related to educating marginalized such as foreign-born communities disproportionately impacted by hepatitis B virus (10–13). Previous health communication campaigns in the U.S. have demonstrated success when utilizing IPC to raise awareness of and improve screening rates for hepatitis B and liver cancer among highly impacted communities (14, 15). However, many of these campaigns have not been informed by the cultural experiences of community members, and their efficacy has not been formally evaluated due to limited resources and organizational capacity (15, 16).

Incorporating IPC into a culturally tailored public health campaign can bring vital information to diverse API, African, and Caribbean communities that are highly impacted by hepatitis B and liver cancer in the U.S. These efforts should be informed by key community leaders to identify effective communication strategies that are culturally appropriate for each distinct community. The need to emphasize the causal relationship between hepatitis B and liver cancer is of utmost importance due to the low levels of existing awareness within heavily impacted communities (17–19). Addressing this connection in a culturally sensitive and community-informed manner can also help remove misperceptions that perpetuate stigmatizing beliefs around these two conditions and promote health behaviors that will help foster early disease detection and management.

This manuscript provides insight into the interpersonal communication messages and channels preferred by foreign-born people living with hepatitis B. It evolved from a broader study that sought to identify current knowledge and awareness about the link between hepatitis B and liver cancer, as well as communication preferences, and access-to-care challenges within various foreign-born groups living in the U.S. Study results can be used to guide the creation of a culturally tailored health education campaign that underscores the relationship between liver cancer and hepatitis B.

## 2 Materials and methods

### 2.1 Data collection

Focus groups and key informant interviews were conducted virtually throughout the United States from April through September 2021. Participants included foreign-born people from API, Haitian, and African communities. Participants were recruited through local community leaders, and a purposive sampling strategy was used to ensure that each community was represented. Sociodemographic information was collected prior to the focus group discussion.

At the start of the project, an advisory committee was assembled with members from the API, African, and Caribbean communities. The committee was composed of public health professionals, healthcare providers, people with lived experience of hepatitis B, and representatives of community-based organizations from around the country. Committee members all had extensive knowledge of hepatitis B and/or liver cancer. This committee contributed to and guided the research process by participating in two focus groups of their own (one with API members and another with African and Caribbean members) and providing insights and perspectives into the challenges of addressing hepatitis B and liver cancer in these highly impacted communities. Committee members assisted in identifying community leaders and members to facilitate and participate in the various focus groups, ensuring that the sample of participants was robust and diverse. The communities selected for this study are communities with which the researchers have existing and highly developed relationships. The community organizations that serve these groups are knowledgeable about the impact of HBV/HCC on these communities and were enthusiastic to participate in this study.

A focus group guide was developed in collaboration with the advisory committee to ensure cultural relevance. These guides were translated into preferred languages by a certified translation company (Elite TransLingo) and reviewed by bi-lingual researchers before use. The guides helped to ensure consistent data collection to allow for accurate comparisons during the analysis phase.

Fifteen focus groups and two key informant interviews were conducted. Focus groups were held with the API and African and Caribbean Advisory Committee members, as well as Micronesian, Chinese (one Mandarin-speaking and one Cantonese-speaking), Hmong, Vietnamese, Korean, Somali, Ethiopian, Filipino, Haitian, and Francophone West African communities. Two different focus groups were held with Nigerian and Ghanaian communities in different geographical locations in the United States. Also, two key informant interviews were conducted with advisory committee members unable to attend the focus group sessions. Focus groups were facilitated virtually over Zoom. Focus group members resided in cities around the United States, including Sacramento, Minneapolis, Seattle, Honolulu, San Diego, Los Angeles, New York City, Philadelphia, Miami, Washington D.C., Chicago, and Boston. All focus groups consisted of seven to 12 participants. The focus groups were recorded and conducted by trained interviewers in either English or another preferred language, determined by the focus group leader. Languages other than English that were used for focus group facilitation included Cantonese, Haitian Creole, Yoruba, French, Amharic, Vietnamese, Marshallese, and Korean. After each focus group, the audio recordings were transcribed and translated into English, if needed. Transcriptions and translations were completed professionally by DataGain Services.

#### 2.1.1 Confidentiality

All focus groups were conducted confidentially, and no identifying information was collected. Verbal consent was collected from all participants as well as verbal authorization to record the focus group and include data in this study’s analysis. The voluntary nature of participating in and contributing to any portion of the discussion was made explicit at the beginning of each group. IRB approval was obtained before the initiation of this study (Heartland IRB Project No. 329-062421).

### 2.2 Data analysis

NVivo 20 software was used for thematic coding and analysis of data. The research team developed a codebook *a priori* that was revised during the coding process as new themes and subthemes emerged. Each transcript was assigned a primary and secondary coder. Transcripts were coded independently and to ensure inter-coder reliability, primary and secondary coders met and discussed each transcript to resolve discrepancies and make final decisions regarding textual analysis. The final kappa coefficient was 0.78, indicating high inter-coder reliability.

## 3 Results

### 3.1 Participant characteristics

A total of 101 people participated in the focus groups, representing a diverse array of cultures (Table 1). Most participants (91.3%) were foreign-born, and the average length of time spent in the U.S. was 25.31 years. Nearly half of the participants reported a preference for speaking a language other than English (48.9%) (Table 2). Comfort levels with spoken English varied, with 52.2% of participants being able to speak English fluently (Table 3). The average comfort level with arranging a doctor’s appointment (used as a proxy for familiarity navigating the United States healthcare system) among participants was 4.2 (on a scale of 1–5, with 1 being not at all comfortable, and 5 being extremely comfortable). The average perceived severity of hepatitis B among participants was 4.2, on a similar Likert scale, and the average perceived severity of liver cancer among focus group respondents was 4.8 (Table 4).

**Table 1:** Demographic characteristics of focus group participants, 2021.

			Residence *N* (%)		Born in U.S. *N* (%)		
	Overall *N* (%)	Mean age in years^a^	Urban	Suburban	No	Yes	Mean length of time in U.S. in years
	101 (100)	52	84 (83.3)	16 (16.7)	92 (91.3)	8 (8.7)	25.3
**Community group**							
Chinese (Cantonese)	6	66.5	6 (100)	–	6 (100)	–	38.5
Chinese (Mandarin)	6	37	3 (50)	3 (50)	5 (83)	1 (17)	22
Filipinx	8	65	8 (100)	–	8 (100)	–	39.5
Hmong	6	36	3 (50)	3 (50)	1 (17)	5 (83)	23
Korean	9	55	9 (100)	–	9 (100)	–	19
Micronesian	5	45	5 (100)	–	5 (100)	–	24
Vietnamese	10	58	10 (100)	–	10 (100)	–	26
Ethiopian	6	54	3 (50)	3 (50)	6 (100)	–	32
Nigerian	9	62.5	9 (100)	–	9 (100)	–	35
Nigerian and Ghanian	6	53	2 (33)	4 (67)	6 (100)	–	18
West African	13	49	13 (100)	–	13 (100)	–	12
Somali	8	41.5	8 (100)	–	7 (87.5)	1 (12.5)	20
Haitian	9	58	9 (100)	–	9 (100)	–	20

a Participant age was not asked directly in any focus group. Instead, age was determined by accounting for age upon arrival in the United States and length of time spent in the United States.

A total of 101 people participated in the focus groups. Most participants were foreign-born and spent an average of 25 years in the United States.

**Table 2:** Spoken language preferences by community group.

	Language preference		
Community group	Prefer speaking English *N* (%)	Prefer speaking a language other than English *N* (%)	Prefer speaking a combination of English & another language *N* (%)
Chinese (Mandarin)	6 (100%)	–	–
Nigerian and Ghanaian (D.C.)	1 (16.67%)	1 (16.67%)	4 (66.67%)
Filipinx	4 (50%)	1 (12.5%)	3 (37.5%)
Chinese (Cantonese)	2 (33%)	4 (67%)	–
Micronesian	–	2 (40%)	3 (60%)
Hmong	5 (83%)	–	1 (17%)
Korean	–	9 (100%)	–
Nigerian (Chicago)	1 (11%)	3 (33%)	5 (56%)
West African	3 (23%)	10 (77%)	–
Ethiopian	2 (33%)	3 (50%)	1 (17%)
Somali	1 (12.5%)	4 (50%)	3 (37.5%)
Vietnamese	–	10 (100%)	–
Haitian	1 (11%)	8 (89%)	–
**Total**	**26 (28.71%)**	**55 (48.86%)**	**20 (22.44%)**

Most participants preferred speaking a language other than English, others preferred speaking English, and some preferred speaking a combination of both English and another language.

**Table 3:** Comfort with spoken English by community group.

	English comfortability			
Community Group	Spoke English fluently *N* (%)	Mostly comfortable speaking English *N* (%)	Somewhat comfortable speaking English *N* (%)	Not at all comfortable speaking English *N* (%)
Chinese (Mandarin)	6 (100%)	–	–	–
Nigerian and Ghanaian (D.C.)	4 (67%)	2 (33%)	–	–
Filipinx	7 (87.5%)	1 (12.5%)	–	–
Chinese (Cantonese)	2 (33%)	1 (17%)	3 (50%)	–
Micronesian	4 (80%)	–	1 (20%)	–
Hmong	5 (83%)	1 (17%)	–	–
Korean	–	2 (22%)	2 (22%)	5 (56%)
Nigerian (Chicago)	5 (62.5%)	1 (12.5%)	2 (25%)	–
West African	2 (15%)	3 (23%)	7 (54%)	1 (8%)
Ethiopian	4 (66%)	1 (17%)	1 (17%)	–
Somali	6 (75%)	1 (12.5%)	1 (12.5%)	–
Vietnamese	1 (10%)	1 (10%)	6 (60%)	2 (20%)
Haitian	–	2 (22%)	6 (67%)	1 (11%)
**Total**	**46 (52.23%)**	**16 (15.27%)**	**29 (25.19%)**	**9 (7%)**

Most participants reported being able to speak English fluently, a smaller percentage of participants were somewhat comfortable speaking English, followed by mostly comfortable and not at all comfortable speaking English.

**Table 4:** Comfort Arranging Doctor’s Appointment, by Community Group.

Community Group	Comfort level arranging doctor’s Appt* Mean (SD)	Perception of hepatitis B severity Mean (SD)	Perception of liver cancer severity Mean (SD)
Chinese (Mandarin)	5 (0)	4 (1)	5 (0)
Nigerian and Ghanaian (D.C.)	4.4 (1.1)	4 (1.1)	5 (0)
Filipinx	5 (0)	5 (0.5)	5 (0.35)
Chinese (Cantonese)	4 (0.9)	5 (0.5)	5 (0)
Micronesian	5 (0.9)	4 (0.5)	5 (0)
Hmong	5 (0.4)	3 (1.3)	5 (0)
Korean	2 (1.33)	4 (0.66)	5 (0.44)
Nigerian (Chicago)	4 (1.33)	5 (1.33)	5 (1.33)
West African	4 (1.4)	4 (1.3)	4 (1.5)
Ethiopian	5 (0)	4 (0.5)	5 (0.4)
Somali	5 (0.9)	5 (0.38)	4.5 (0.38)
Vietnamese	3 (0.99)	3 (1.2)	4 (1.3)
Haitian	3 (1.4)	4 (1.1)	5 (0.33)
**Total**	**4.18** (0.988)	**4.15** (0.688)	**4.81** (0.38)

The average comfort level with arranging a doctor’s appointment was used as a proxy for familiarity navigating the United States healthcare system. The responses are based on a Likert scale, with 1 being not at all comfortable and 5 being extremely comfortable. The average comfort level arranging a doctor’s appointment among participants was 4.2. On a similar Likert scale, the average perceived severity of hepatitis B and liver cancer among participants was 4.2 and 4.8, respectively.

### 3.2 Qualitative findings

#### 3.2.1 Explaining the link between hepatitis B and liver cancer

Among the API, African, and Haitian communities, all agreed that communication must clearly explain the connection between hepatitis B and liver cancer. One member of the Chinese Cantonese-speaking community asked the moderator to *“…explain what’s the difference between hepatitis and liver cancer.”* Similarly, one Filipino participant shared that there is a lot of confusion within their community regarding how hepatitis B occurs and how it progresses to liver cancer. A participant in the Haitian focus group discussed their understanding of how hepatitis B may lead to liver cancer: *“Well, what caught my attention as to when this disease degenerates and turns into cancer”.*

It is also necessary to inform the community about the benefits of screening and management of hepatitis B, as these behaviors will reduce the risk of liver cancer. When asked by the interviewer, a key informant agreed that messaging should emphasize how hepatitis B can put people at increased risk for liver cancer, as this would make people more likely to get screened for both conditions. Francophone West African, Hmong and Haitian focus group participants voiced opinions related to the importance of messages that draw a clear connection between the diseases, and to their belief that testing will increase with knowledge.

#### 3.2.2 Identified interpersonal communication preferences

##### 3.2.2.1 Preferred messenger

Across multiple communities, the source of health information was often identified as holding more importance than the message itself. Influential messengers included local healthcare providers, community health workers, and community and religious leaders. Focus group participants agreed that messengers must be trusted, have a shared culture with the audience, and speak the native language of the community. A Vietnamese participant explained, *“We encourage doctors to share information because people do not listen to others but to doctors. Many older people normally debate a lot with others but usually agree with doctors.”* Other focus group participants, including African and Caribbean members of the advisory committee, and the Somali, Micronesian, Francophone West African, and Hmong communities also discussed the importance of having a trusted source to influence community members, disseminate messages, and encourage people to seek hepatitis B screening and care. For instance, a Micronesian participant stated, *“When we work with people from the community, they know who to speak to, and they know what the crowd is comfortable with, and the level of understanding from our community.”* A Somali participant further emphasized their community’s acceptance of messages when, *“…the message is given by somebody that they trust, that they know, who speaks their language, who understands their culture”.*

A Mandarin-speaking participant shared the importance of collaborating with faith leaders when creating messages: *“If we were to reach out to certain religious groups, I think it’s good to touch base with maybe their leaders…to check what message to not give, or things to avoid.”* For some communities, the gender of the messenger also mattered. One Micronesian community member stated, *“There are some things that men only say amongst men, women only say amongst women.”* A Somali participant also asserted, *“If you are a woman, women should be teaching the class or training. If there’s a male, a male should be comfortable”.*

##### 3.2.2.2 Preferred language

Across every focus group community, English was cited as being more accessible for younger generations, while older generations often prefer to receive health messaging in their native languages. Ideally, campaign activities should be conducted in both English and the community’s native language. One of the key informants explained how he presents information to the Ghanaian community:

*I try to speak English, but then I try to also speak the local dialect that I think most people understand and speak to them about what I’m trying to say or what the important message is. So, I use both.*

A Nigerian community member also provided insight into their community’s language preferences, especially regarding verbal and written information:

*Maybe you want to talk to them in a seminar form, maybe like in the mosque setting, I think it’s where you speak Yoruba, then maybe when you are passing like a flyer that you can pass to people English will be more appropriate.*

##### 3.2.2.3 Preferred setting

Community-based settings, including social clubs, and local churches and mosques, were identified as key places to reach entire groups of people. As a Cantonese-speaking participant said, *“You need to hold seminars and invite the Asian communities to attend them. Why? Because general public do not know about hepatitis antibody, cirrhosis and the differences between these diseases.”* A Hmong participant expressed their thoughts on the importance of interacting in settings where the community feels safe:

*I would really, certainly encourage this information to be given in a place where there’s already Hmong people feeling really comfortable. And they know ahead of time that it’s going to be there, and again, putting it in an atmosphere that allows for a communal feel.*

In keeping with this theme, an Ethiopian community member shared, *“I think churches are very powerful so, if we do it, maybe using the church platform, because they still attract many people.”* Haitian, Francophone West African, Nigerian, and Mandarin-speaking focus group participants, as well as one of the key informant interviewees, echoed these opinions. For the Hmong community, face-to-face communication was felt to be more influential than other communication methods. In the words of one participant: *“Well, I have questions. ‘Who should I talk to?’ I do not know. To me, I just like to speak with people* versus *just clicking a link”.*

Healthcare settings were also noted as important and trusted places for dissemination of health information and education. A Cantonese-speaking member shared, *“I think you need to get hospitals to be involved in this. Otherwise, people will not pay attention. They will be like, ‘Why should I get these shots?’.”* A member of the Nigerian and Ghanaian focus group explained, *“If the doctors … can explain to me clearly as to why this is important, or this is necessary to take, I will take it.”* Nigerian participants highlighted the need for multiple channels for interpersonal communication. One suggested that doctors should have materials in their offices, *“not just outside in the waiting area, but when they are in the room. And it’s even better if a doctor could hand it to* [their patients].” Another added that receiving information from the doctors is:

*Going to be helpful, but at the same time, it’s going to be only for the people that go to the doctor’s office -if I do not go to the doctor’s office, I will not know that something like that is there.*

##### 3.2.2.4 Preferred messaging channels

The accessibility, ease, convenience, and wide reach of mobile messaging applications, including WhatsApp and its equivalents, was noted by various groups. Members in the API advisory committee mentioned using messaging applications to reach different communities, including Zalo for the Vietnamese community, Kakaotalk for the Korean community, Line for both the Japanese and Taiwanese communities, and Clubhouse and WeChat for the Chinese community. Similarly, an African advisory committee member stated *“…the only way that we communicate is through WhatsApp.”* A key informant interviewee posited, *“What I know is everybody, I think a lot of Africans use WhatsApp.”* Members of the Somali and Ethiopian groups also noted that most people in their communities use WhatsApp, and a Somali participant recommended that educational campaigns use the application to disseminate information. They added that this platform can also increase the likelihood of older people interacting with the campaign, as they often *“…do not have access to social media besides WhatsApp”.*

However, some community members, such as one in the Hmong focus group were apprehensive about the use of messaging applications to disseminate educational materials, considering them to be *“too intrusive.”* A Nigerian participant mentioned that if they were sent educational information through a messaging platform from an unknown messenger instead of speaking to a trusted community member, they would *“just push it away,”* reinforcing the need for trusted messengers to deliver health information.

Personal testimonials were identified as a meaningful way to reach many communities. One key informant stated, *“When I do education, sometimes I talk about my story. I talk about my experience, but then sometimes I also bring stories that other people have shared with me.”* A Hmong participant discussed how personal testimonials can help destigmatize health topics:

*I think you got to have a person like me go out to the community and say, ‘Hey, look at me, I’m like you. I have it, here’s why I have it…here’s my story…And here’s what it leads to’.*

#### 3.2.3 Communication themes

##### 3.2.3.1 Hope versus fear

Overall, participants across all focus groups preferred informational messages to be hopeful and empowering. A Nigerian participant shared, *“When someone projects fear, it would make you feel like you cannot do anything about it. But when you use the message of hope, the person would feel that at least, something can be done about it.”* A Francophone West African community member explained that “…*the more aggressive it is, the more discouraging it will be, and people withdraw”* and emphasized that *“softer, understandable speech”* is more accepted within their community. Similarly, a Hmong participant articulated, *“I do not think that fear is going to help drive grandpas and grandmas to go and get tested”*.

However, participants in the Mandarin-speaking, Korean and Vietnamese communities proposed that communicating information using both hopeful and fearful tones could prove useful in their communities. A Korean participant shared their thoughts on the benefits of including messages that instill some fear in the audience to promote behavior change: *“It would be better if it included a message about fear and alertness about this disease because many people have heard of hepatitis B but do not know exactly what it is”*.

##### 3.2.3.2 Quality of life

Multiple communities discussed the importance of messages that focus on one’s quality of life. A Hmong participant mentioned their thoughts on how a hepatitis B diagnosis is not the end of one’s life: *“I think having the fact that you can still get married and have children, that there’s still life after being diagnosed with hepatitis B or having liver cancer.”* Others agreed on the need to include quality-of-life messages. One key informant interviewee described the importance of not just providing information but also, “…*reassurance and comfort that whatever the result, things will still continue as usual, and so information is important, but it should be supplemented with reassurance”*.

##### 3.2.3.3 Respect

Community members discussed the need for respect for social and cultural norms within campaign communications specifically about sexual health, given that this is one possible transmission route for hepatitis B. A Micronesian participant articulated:

*When we do community outreach in the Chuukese community, it’s very disrespectful to talk about the sexual like, just even mentioning, the different parts of man and woman or like anything that only the men say among themselves or the lady say among themselves.*

Respect related to discussing community-specific risk for hepatitis B and liver cancer was also routinely mentioned, although there were differences within and among communities. Vietnamese, Nigerian, Hmong and Micronesian community members shared that when presenting the risk to their specific community, such messaging should be grouped with other populations that are also at risk. While one Francophone West African community member advised against specifically naming communities which are heavily impacted by hepatitis B, another individual from the same group requested that information explain the reality of who is most affected by the disease. The idea to outline how hepatitis B can be more prevalent among certain populations in campaign communications was supported by the Ethiopian and Mandarin-speaking communities as well.

A participant in the Nigerian and Ghanaian group discussed the need to respectfully acknowledge the misconceptions that lead to personal stigma through education and statistics:

*Well, definitely education is a better approach, because you are going to give them a background and a history that they did not do something dirty to get it, right? Perhaps you draw the attention to it, that it’s nothing that they did just that they were born that way, or because of the socio-economic reasons, and based on studies, and we have this percentage or this number, you have facts, something more tangible to tell a person, and they may not have been aware, but letting them know that it’s more probable that many people in your community have it, but it’s unknown, so they are not alone in this.*

##### 3.2.3.4 Community

Resources highlighting an individual’s role within their community were cited as universally important. A Haitian participant described the importance of calling upon the community to increase social support: *“We have to inform the people around them not to isolate them, on the contrary to help them. Bring consciousness to all the community.”* Micronesian participants also emphasized how campaigns should employ a holistic approach:

*People with increased risk for liver cancer, we need to do a lot of again education and other kinds of psychological, social help, or spiritual help even for some folks…they need some sort of support system in order to maintain their healthier lifestyle to reduce their risk as much as possible*.

Additionally, messaging that emphasizes and incorporates one’s irreplaceable role in their family is considered important among most communities. An API advisory committee member voiced this idea, *“We do not like to do screening or any testing. But if it comes down to this will help your family, then people were more willing and wanted to do it.”* A Vietnamese community member articulated how taking care of themselves is their responsibility to their family. Similarly, one key informant expressed, *“With almost every African, family is very important to us. So, maybe to draw on that idea that if you take care of yourself, then you will be there to take care of your family”*.

##### 3.2.3.5 Religious messaging

Perspectives on religious messaging varied. A Somali participant recalled a positive experience with a faith leader discussing public health issues: *“*[He was] *very encouraging and he had like some quotes from the Quran when he was saying public health is like religious duty and then we all have the responsibility to care for one another.”* A Francophone West African participant also defined the relationship between their Muslim faith and their health behaviors:

*As a faithful Muslim everything is there. Islam asks you to take care of your health because you must be healthy to praise. Even the Prophet, when he was sick, he had to go and see the doctor. In the message, you can show that everyone gets sick. Even our Prophet… Go get treated.*

While some communities had positive perceptions towards faith-based messaging, most requested that messaging remain science-based. A Nigerian participant advised, *“Just be specific about the health issue, nothing about — this is not about religion issue at all.”* A Vietnamese community member stated, *“I think our information relates to science, medicine, and medical knowledge, it has nothing to do with religions or beliefs, we should talk only about science, we have no reason to mention about religions or beliefs.”* Other respondents disclosed how some people in their community would respond negatively to religious messaging. For instance, a Hmong participant said, *“Religion stuff is really touchy. And you really push away a lot of people, prospective people that would really benefit from the information.”* The Cantonese-speaking, Vietnamese, and Filipino focus groups agreed that faith-based messaging should be avoided for similar reasons.

## 4 Discussion

This study’s exploration of the similarities and differences in interpersonal communication preferences across various immigrant communities underscores the vital nature of culturally appropriate approaches for the dissemination of critical health messages and the promotion of health-protective behaviors. These findings are supported by existing literature, and align with established theories of interpersonal communication (9). They not only provide additional insight into how health communication needs compare across communities, but also examples of how to tailor direct messaging and educational presentations to meet the social and cultural expectations of individual communities, as well as their health literacy levels (20).

### 4.1 Communication preferences

#### 4.1.1 The importance of the messenger

This study found that across all communities, the person delivering a health message holds equal importance as the message content. Additionally, this study and others found that the knowledge and expertise of healthcare providers make them a credible source of health information and an ideal messenger (21, 22). Campaigns that invite hepatitis B and liver cancer experts or family physicians to inform the priority population can increase the audience’s acceptance of the information, as trust in doctors is high, especially when they share a language in common with the audience (17, 23).

Somali, Micronesian, Francophone West African and Hmong community members in this study also discussed how they trust people who are endorsed by their community leaders, such as public health practitioners and researchers, especially when they understand and respect the community’s cultural nuances. Community health workers, patient navigators, and lay health educators who are knowledgeable about liver health and share the audience’s language and culture can promote acceptance of an educational campaign within a given community and overcome cultural and linguistic barriers (24–26).

Messengers should also reflect the gender of the intended audience, especially when discussing sexual transmission of hepatitis B (26). In the present study, the Ethiopian, Mandarin-speaking, Micronesian and Somali communities emphasized the need for the audience to receive information presented by a person of the same gender. A previous study found that participants showed improved hepatitis B-related knowledge and higher acceptance of an awareness campaign because male staff conducted the study procedures with men while female staff facilitated program activities with women (26). Additionally, other research has found that African-born women often seek health information from their female social circles, as they are able to openly converse about their health concerns and relate to others’ health experiences (21).

Religious leaders have been found to be particularly effective in conveying health information in communities with lower literacy levels, as they have developed strong and understanding relationships with community members and older generations (27). This has been demonstrated in other programmatic efforts: Hepatitis B-related knowledge increased among West African-born participants who received educational content that was informed and reviewed by community organizers and faith leaders (28).

#### 4.1.2 The importance of setting

Ideally, interpersonal communication should take place across multiple settings. Prior to campaign implementation, public health professionals must build connections with community organizations by attending in-person events to establish a relationship with the community and eventually increase audience engagement.

Educational seminars should be held in places that people frequent, including social clubs, healthcare settings, and faith institutions. By meeting communities where they feel most comfortable, messengers can establish a trusting relationship with the audience. For example, previous research has found that holding educational presentations in non-traditional settings, such as an English as a Second Language course can increase community engagement and can prove useful at reaching foreign-born populations who may benefit from learning about hepatitis B and liver cancer (29). Community-based organizations are vital spaces that provide health-based activities to community members and are easily accessible and highly trusted. These organizations have been found to be especially important when educating older populations (30).

Communication campaigns that take place in healthcare settings can reach both patients and healthcare providers, as providers themselves are considered trusted sources of information in many communities. By ensuring that trusted providers have complete and accurate knowledge about the connection between hepatitis B and liver cancer, their ability to serve as effective ambassadors and educators for their communities will only improve (31). People in the Korean and Cantonese-speaking communities highlighted that healthcare settings are favored places for information exchange, as there is opportunity to ask questions while learning about hepatitis B. However, it is necessary to account for age when determining a campaign’s location, especially since older populations often have difficulty accessing formal health services due to linguistic barriers, transportation challenges, and not knowing where to seek care (30).

Members of the Ethiopian, Haitian, Francophone West African, Nigerian, Ghanaian, and Mandarin-speaking communities all agreed that religious establishments are places where people feel comfortable and willing to interact with a campaign’s health information and seek further opportunities to access care (32). This counters the findings from a previous study, where African-born women stated that while the church is a source of social support, they do not turn to the church for health information, as diseases such as cancer are not discussed due to associated stigma (21). Caution should be exercised when implementing a campaign in faith-based settings, especially when determining whether the iteration of fatalistic beliefs and disease-related stigma is appropriate for different communities (21, 32).

Additionally, thoughts about the utility of incorporating religious concepts and ideas into educational materials varied across communities. Although participants recommended the use of religious spaces to host educational initiatives, all but a Somali participant recommended against messages that referenced religion. Since hepatitis B is not considered a religious issue, the Nigerian, Hmong, Vietnamese, Cantonese-speaking, and Filipino communities preferred that informational materials avoid specific religious messaging within the health content.

#### 4.1.3 The importance of delivery

The Vietnamese, Francophone West African, Somali, Haitian, Hmong, Nigerian, Ghanaian and Filipino communities all agreed that information should be presented in both English and in identified native languages. Use of an audience’s native language is an essential component of culturally tailored interventions, especially when educating older groups (11, 30). Educational content should be customized to specific age groups within the priority population, as older people tend to communicate in their native language and follow cultural practices more often than their younger counterparts, who prefer to communicate in English. Older generations also often rely on doctors, faith leaders, word of mouth, and people in their social circles to learn about health information. It is also important to tailor themes of educational content to age, as older people tend to be less receptive to fear-based messaging than younger people (23). Communicating information to an audience in their native language enhances understanding of the content and helps practitioners gauge what topics need to be reinforced.

Verbally conferred health information, including personal testimonials and storytelling, was identified as a powerful way to disseminate hepatitis B-related information in both this study and prior research (23). When tailoring health information to a specific audience, it is necessary to account for the way information is traditionally presented in the audience’s culture. For example, West and East African cultures often share information through verbal exchange, especially in cases where a language is not typically written (21, 28). Verbal communication methods may be more effective at reaching African-born populations, especially women, who have traditionally learned through generational storytelling (21, 28). Moreover, in the present study, the Micronesian, Somali and Hmong communities all expressed how personal testimonials can destigmatize topics, combat racism, and enhance a community’s acceptance of a message that is delivered through a relatable storyteller.

Mobile messaging applications, such as WhatsApp, WeChat, and equivalents used in different countries have also been identified as useful channels for sharing instructional videos and informational health messages to broader social circles (14, 33). As these applications are digital, information can easily be delivered in an audience’s preferred language, and the platforms allow for immediate communication between the audience and health educators. This method is most impactful when video links and educational messages are sent by trusted community members or faith leaders, as they increase peoples’ acceptance of the information.

With all communication channels, it is vital to understand and incorporate the distinct cultural nuances and health literacy levels within each community group (20). Additionally, when communicating disease-related information to community members, health educators should integrate hopeful messages into the campaign by reminding people that hepatitis B does not have to severely disrupt their quality of life, as there are effective ways to maintain health and reduce risk of liver cancer (22). Audience members may be more receptive to disease-related information when educators focus on how an individual is protecting their family and greater community by practicing hepatitis B and liver cancer prevention behaviors. Encouraging social support and healthy lifestyles during these conversations is another way to positively frame prevention behaviors.

Ensuring that the presented information does not further stigmatize certain groups is essential when educating communities heavily impacted by hepatitis B and liver cancer. When possible, education programs can inform the audience of their risk without placing specific blame on their community by grouping impacted populations together, as voiced by members of the Vietnamese, Nigerian, Hmong and Micronesian communities. Programs should also explain why and how the information is relevant to their community, as suggested by members of the Francophone West African, Ethiopian, and Mandarin-speaking groups. Some participants in the Mandarin-speaking, Vietnamese, Korean, Ghanaian and Nigerian communities also expressed the need for information that emphasizes the potential danger of hepatitis B and liver cancer, as fear can motivate individuals to manage their hepatitis B and practice liver cancer prevention behaviors. Whether focusing on positivity or incorporating risk language, all educational conversations and presentations must remind the audience that they can manage their hepatitis B to live a healthy life and lower their risk of liver cancer (22). Emphasizing the need for continuous monitoring and management reinforces the connection between hepatitis B and liver cancer and calls on the audience to maintain their health (34).

Finally, messages must emphasize that hepatitis B is a direct cause of liver cancer, especially if left undiagnosed or unmanaged (7). This was specifically requested by members of the advisory committee, and Filipino, Francophone West African, Hmong, and Haitian groups. In the present study, both the Ethiopian and Mandarin-speaking communities asked that educators explain to their communities how hepatitis B screening and ongoing management can reduce one’s risk of liver cancer. By framing hepatitis B screening as a liver cancer prevention strategy and highlighting the connection between the two diseases, community members may become more motivated to get screened and recognize the health benefits of engaging in these behaviors, as found in previous research (13).

### 4.2 Limitations and lessons learned

This study has several limitations and lessons learned. Given the constraints inherent in qualitative research concerning generalizability, the results from this study are not generalizable to all immigrant communities in the U.S. due to variations in cultural norms, linguistic diversity and health literacy levels. However, it is not the intention to generalize results to the broader U.S. population but rather provide situated findings. Despite employing a purposive sampling strategy for participant recruitment and rigorous data collection, selection bias may pose a threat to the study’s transferability as demographic diversity may have been limited within each community focus group. Further outreach strategies could be employed to include a broader range of perspectives from subgroups within the focus group communities, as well as other immigrant communities in the U.S. However, the study results are still transferable to other populations with similar characteristics, such as other foreign-born groups with increased risk of hepatitis B.

Social desirability bias may also impact the credibility of the study results, as the focus group setting may have prompted sharing behaviors that may have differed from those exhibited in individual key informant interviews. Some focus group participants were providers themselves who had existing knowledge about hepatitis B and liver cancer, which could have impacted the natural course of group discussions. In this study, one focus group conversation was subject to leading questions and prompts posed by a facilitator, so participants’ answers may not have been organic. Additionally, as the focus groups were conducted over Zoom, prospective participants who have low digital literacy or do not have access to the required technology may have been excluded. Future efforts can plan to host both in-person and online focus group sessions to account for this limitation.

This study may have been subject to challenges with translation. Focus groups that were conducted in a language other than English were professionally transcribed and translated; however, there may have been some missed jargon or incorrect translations of words or phrases. In addition to professional translation, and review of the translations by community members who speak the language, further validation measures for translated materials, and in-language moderator training sessions could mitigate potential biases.

This study found that the focus groups themselves were often a source of awareness and knowledge for participants. In the future, a small educational session following each group or the provision of a fact sheet or other resource for participants would be beneficial. The researchers will plan to include an assessment of knowledge, behaviors and attitudes after exposure to educational materials, in tandem with the dissemination of the communication campaign that was informed by, and is following, this study. This will be an effective tool for capturing the impact of newly acquired knowledge on behavior.

## 5 Conclusion

This study identified culturally specific IPC preferences for hepatitis B and liver cancer messaging within diverse communities. Communication preferences varied in many ways between different groups, but overall, we learned that we need to directly address prevailing culturally specific misconceptions around hepatitis B and liver cancer by presenting the facts around the diseases and their connection. Participants agreed that health messaging must emphasize that hepatitis B is not a death sentence, and explain how screening and vaccination, and ongoing care and treatment for those with hepatitis B, can keep people healthy. Participants also agreed that messaging should be delivered by trusted messengers who share ethnicity, gender, language, and even age with the audience, in settings where communities feel safe. The importance of culturally appropriate messaging was emphasized, as there were community-based differences surrounding the use of hope and fear, religious messaging, and delivery channels, confirming that any communication materials developed must be community-specific to be effective. Learnings from this study can lay the groundwork for effective educational programming that focuses on liver cancer prevention, to promote uptake of hepatitis B screening, vaccination and linkage to appropriate care among the most highly impacted communities in the U.S.

## Data availability statement

The original contributions presented in the study are included in the article/Supplementary material, further inquiries can be directed to the corresponding author.

## Ethics statement

The studies involving humans were approved and received ethics approval through Heartland IRB (HIRB project no. 329-062421). The studies were conducted in accordance with the local legislation and institutional requirements. The participants provided their verbal informed consent to participate in this study.

## Author contributions

BZ: Conceptualization, Data curation, Formal analysis, Investigation, Project administration, Writing – original draft, Writing – review & editing. SB: Data curation, Formal analysis, Investigation, Writing – original draft, Writing – review & editing. FB-J: Writing – original draft, Writing – review & editing. KM: Conceptualization, Data curation, Formal analysis, Investigation, Methodology, Writing – original draft, Writing – review & editing. TC: Investigation, Writing – original draft, Writing – review & editing. RA: Investigation, Writing – review & editing. DH: Investigation, Writing – review & editing. CC: Conceptualization, Funding acquisition, Project administration, Supervision, Writing – original draft, Writing – review & editing.
