# Supplementary material for: The role of culturally appropriate interpersonal communication strategies to reduce hepatitis B and liver cancer disparities
Source: Front Public Health. 2024 Aug 9;12:1377096. doi: 10.3389/fpubh.2024.1377096 (PMC11341449; doi:10.3389/fpubh.2024.1377096)
Supplement: Supplementary file 1 [file Data_Sheet_1.docx]

Supplementary Material

The Role of Culturally Appropriate Interpersonal Communication Strategies to Reduce Hepatitis B and Liver Cancer Disparities

Beatrice Zovich, Suzanne Block, Fiona Borondy-Jenkins^*^, Kate Moraras, Thomas Chen, Rukayat Adedokun, Dung Hua, Chari Cohen

*** Correspondence:** Fiona Borondy-Jenkins: [fiona.bjenkins@hepb.org](mailto:fiona.bjenkins@hepb.org)

[Supplementary Material Guidelines](https://www.frontiersin.org/guidelines/author-guidelines#supplementary-material)

# Supplementary Figures and Tables

## Supplementary Figures


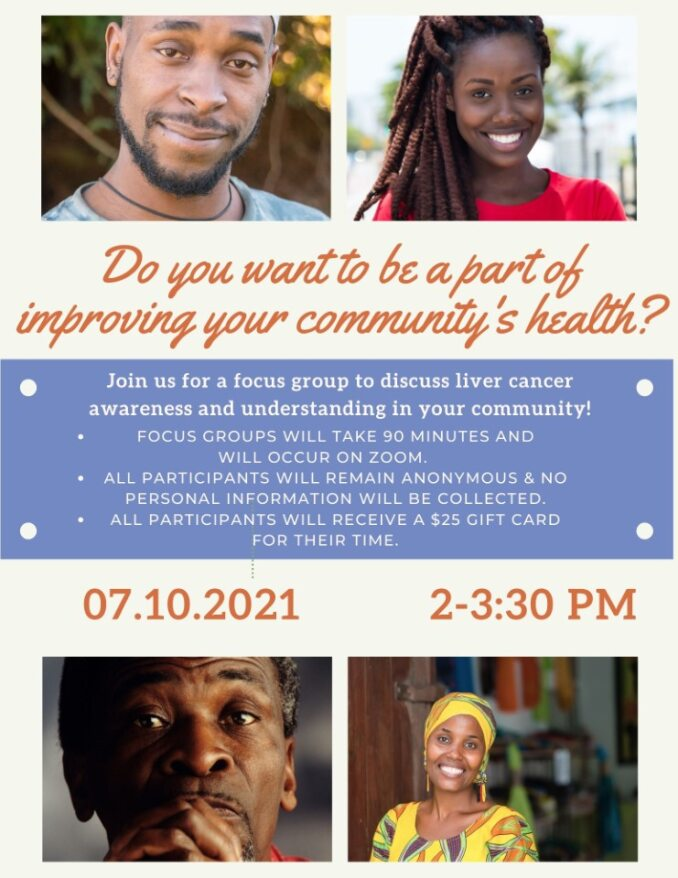


**Supplementary Figure 1.** The above flyer was used to recruit potential participants for this study.

## Supplementary Figures


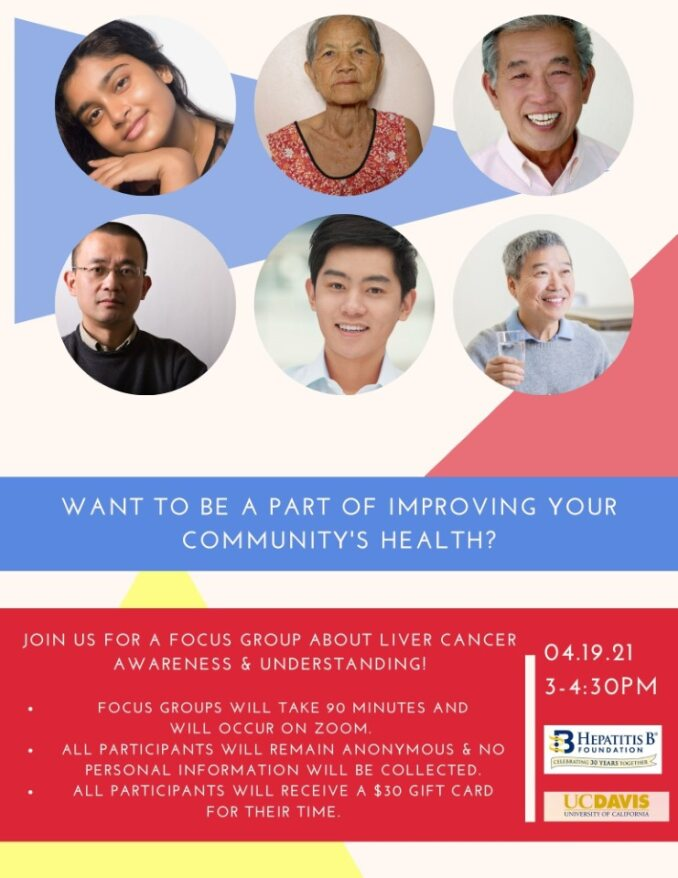


**Supplementary Figure 2.** The above flyer was used to recruit potential participants for this study.

## Supplementary Figures

**Focus Group Facilitator Guide**

Thank you so much for stepping into the important role of being a focus group facilitator! Your contribution to the research process and to community health is an important one. Leading a productive focus group can be incredibly rewarding - and also a bit challenging. Below, please find some guidelines that can help in the process. Thanks so much again for your assistance!

- The most important aspect of facilitating a focus group is maintaining control of the conversation. **This means doing your best to ensure that everyone’s voice is heard and that everyone feels comfortable speaking, but no one dominates the conversation.** It’s a balance! There is an icebreaker question included in the focus group guide that will hopefully get people comfortable with each other from the beginning.
- At the beginning of the focus group guide, there is a script that introduces the purpose of the focus group, and the basic ground rules. **These rules are very important and will help with guiding the conversation, so it’s very important to please read them to the group and encourage all to follow them all the way through.**
- It may very well be the case that you are an expert in this area and have your own opinions about some of the topics that are covered or questions that are asked during the focus group. **It is very important that you hold back from expressing your own opinions and only stick to the script in the focus group guide.** We want to get honest and organic answers from focus group participants and do not want them to be influenced in any way.
- **Timekeeping**: **The whole focus group should take a total of 90 minutes.** Within the focus group guide, you will notice that next to the heading of each section, there is indicated a time frame for that set of questions. This is not hard and fast, but just something to keep in mind for about how much time each question set should take, in order to keep things on track and moving forward in a timely manner. It might be helpful to set a timer for each set of questions, just to keep things moving forward. If you do get to the 90-minute mark and still have questions left, you can do one of two things. You can either finish up the question you are on, and note the questions that you didn’t get to. Or you can ask participants if they all agree to stay on an additional 10-15 minutes – **but only move forward if everyone agrees to stay on.**
- **Demographic Questions**: At the very beginning of the focus group, participants will be asked to answer some very brief demographic questions. For groups that are occurring in a language other than English, a document with these questions translated into the appropriate language will be shared with you. You can choose to either share this with the group on your screen and have the participants respond verbally or in the chat box, read the questions out loud and have participants respond either verbally or in the chat box, or send the questions to participants either before or after the focus group in order to preserve anonymity.
- **Zoom for Beginners**: This may be the first time that some of the participants in your focus group are using Zoom. Here is an overview of the most basic functions of Zoom to offer to participants at the beginning of the focus group:
  - **Names:** Since we would like the focus group to be as anonymous as possible for research purposes, we would like people to either use their first names only in the Zoom or to use a nickname or pseudonym. Feel free to instruct people that in order to chance their names, they can click in the upper right-hand corner of the square that shows their name and image (where they should see three dots), and after clicking on the three dots, they should see an option that says “Rename,” which they can click on and type in their preferred name and then click OK.
  - Point out the camera button, so people can understand how to turn their video on and off. **In order to facilitate discussion, please encourage people to keep their cameras on as much as possible.**
  - Point out the microphone button, so that people understand how to mute and unmute themselves. As the host of the meeting, you will also have the ability to mute and unmute people as well, in case someone is not able to do it themselves. **Please encourage people to stay muted, unless they are speaking** - this will limit background noise.
  - Point out the chat feature and let people know that they can type thoughts or answers to questions there if they would like. **It is a good idea to keep an eye on the chat throughout the group to address any questions or concerns that might appear there.**
- Below are some YouTube videos that you may find helpful in providing tips and tricks for running a successful online focus group:
  - YouTube Video: <https://www.youtube.com/watch?v=Auf9pkuCc8k> (What to Do and What Not To Do)
  - YouTube Video: <https://www.youtube.com/watch?v=KlCDgVCQRNg> (The Difference Between In-Person and Online Focus Groups)

Thank you so much again for your time and effort in conducting these focus groups. Your help is very much appreciated! For any questions or concerns, please do not hesitate to reach out to Beatrice Zovich at [beatrice.zovich@hepb.org](mailto:beatrice.zovich@hepb.org).

**Supplementary Figure 3.** The focus group facilitator guide was used to prepare people to facilitate the community focus groups in this study.

## Supplementary Tables

**Liver Cancer Disparities Project Codebook**

| **Code** | **Definition** |
| --- | --- |
| **Awareness of hepatitis B** | Use when an individual indicates whether they have heard of hepatitis B and their level of awareness of the existence of the disease. |
| **Awareness of liver cancer** | Use when an individual indicates whether they have heard of liver cancer and their level of awareness of the existence of the disease |
| **Healthcare Utilization** | To be used when a participant indicates a specific type of healthcare that is typically utilized within their community |
| **Insurance Status** | Use when a participant describes the insurance status of a majority of their community members |
| **English Language Proficiency** | Use when an individual describes their community’s level of English language proficiency |
| **Existing hepatitis B knowledge/perceptions** | Use when an individual indicates their level of knowledge about hepatitis B facts (transmission, prevention, diagnosis, treatment, etc.), regardless of accuracy. |
| **Route of hepatitis B awareness or knowledge acquisition** | Use when an individual indicates how they became aware of or obtained knowledge about hepatitis B. |
| **Existing liver cancer knowledge/perceptions** | Use when an individual indicates their level of knowledge about liver cancer facts (transmission, prevention, diagnosis, treatment, etc.), regardless of accuracy. |
| **Route of liver cancer awareness or knowledge acquisition** | Use when an individual indicates how they became aware of or obtained knowledge about liver cancer. |
| **Awareness of liver cancer and hepatitis B connection** | Use when an individual speaks to their level of awareness of a connection between hepatitis B and liver cancer. |
| **Existing knowledge/perceptions of liver cancer and hepatitis B connection** | Use when an individual indicates their level of factual knowledge about the connection between hepatitis B and liver cancer (regardless of accuracy). |
| **Existing awareness about other health conditions** | Use when an individual indicates that they have heard of or are aware of the existence of health conditions besides hepatitis B and liver cancer (such as heart disease, hypertension, diabetes, or other types of cancer). |
| **Existing knowledge/perceptions about other health conditions** | Use when an individual indicates their level of factual knowledge about other health conditions, regardless of accuracy. |
| **Route of acquisition of knowledge/awareness of other health conditions** | Use when an individual indicates how they became aware of or obtained knowledge of other diseases. |
| **Stigma/shame- general** | Use when a participant speaks to stigma and shame and does not specify whether it relates to hepatitis B or liver cancer. |
| **Stigma/shame around hepatitis B** | Use when an individual speaks about stigma or shame specifically associated with hepatitis B. |
| **Stigma/shame around liver cancer** | Use when an individual speaks about stigma or shame specifically associated with liver cancer. |
| **Differences between hep B & liver cancer shame/stigma** | Use when an individual refers to any differences in the type of stigma or shame surrounding hepatitis B or liver cancer. |
| **Barriers and Challenges- General** | Use when a participant speaks to barriers and challenges to access to care or the desire to seek care and does not specify whether it relates to hepatitis B or liver cancer. |
| **Barriers/challenges to hep B prevention/testing/diagnosis** | Use when an individual shares any reason not to take preventative measures, be tested for HBV or a barrier, either personal, institutional, or systemic, to getting tested for HBV. |
| **Hiding status - general** | Use when an individual indicates whether or not they would hide their status or diagnosis or speaks about reasons for doing so for any disease. |
| **Hiding hep B status** | Use when an individual indicates whether or not they would hide an HBV status or diagnosis or speaks about reasons for doing so. |
| **Hiding liver cancer status** | Use when an individual indicates whether or not they would hide a liver cancer status or diagnosis or speaks about reasons for doing so. |
| **Desire for knowledge of hep B diagnosis** | Use when an individual discusses whether or not they would want to know if they were living with HBV and the reasons behind this. |
| **Desire for knowledge of liver cancer diagnosis** | Use when an individual discusses whether or not they would want to know if they were living with liver cancer and the reasons behind this. |
| **Barriers/challenges to liver cancer prevention/testing/diagnosis** | Use when an individual shares any reason not to seek preventative care, to be tested for liver cancer or a barrier, either personal, institutional, or systemic, to getting tested for liver cancer. |
| **Feelings of control over hep B diagnosis** | Use when an individual shares about whether or not they believe a hepatitis B diagnosis is within their personal control and the reasons behind this thinking. |
| **Feelings of control over liver cancer diagnosis** | Use when an individual shares about whether or not they believe a liver cancer diagnosis is within their personal control and the reasons behind this thinking. |
| **Role of fate or inevitability in diagnosis** | Use when an individual shares their feelings about whether or not a hepatitis B or liver cancer diagnosis is destined or inevitable and/or their reasons behind this thinking. |
| **Perceived liver cancer risk from hepatitis B** | Use when an individual estimates how high the risk of liver cancer is for a person living with HBV. |
| **Information/messages/content to Include in an awareness campaign** | Use when an individual shares what types of information, messages, or content would be helpful to include in a communications campaign meant to reduce liver cancer disparities. |
| **Information/messages/content to leave out/avoid putting in an awareness campaign** | Use when an individual shares what types of information, messages, or content would be best to avoid using in a communications campaign meant to reduce liver cancer disparities. |
| **PARENT CODE: Delivery methods for awareness campaign** | Use when an individual indicates the most suitable and appropriate channels through which to deliver an awareness campaign about liver cancer disparities. |
| **SUBCODE: Value of/thoughts around social media usage** | Use when an individual speaks to the utility or lack thereof of social media for dissemination of a communications campaign. |
| **SUBCODE: Value of/thoughts around community health educators/workers** | Use when an individual speaks to the utility or lack thereof of community health educators/workers in disseminating a communications campaign. |
| **SUBCODE: Value of/thoughts around other community or religious leaders** | Use when an individual speaks to the utility or lack thereof of other community or religious leaders in disseminating a communications campaign. |
| **SUBCODE: Value of/thoughts around printed materials** | Use when an individual speaks to the utility or lack thereof of printed materials (found in a doctor’s office or community setting) for dissemination of a communications campaign. |
| **SUBCODE: Value of/thoughts around radio announcements** | Use when an individual speaks to the utility or lack thereof of radio (ethnic or mainstream) for dissemination of a communications campaign. |
| **SUBCODE: Value of/thoughts around podcasts** | Use when an individual speaks to the utility or lack thereof of podcasts for dissemination of a communications campaign. |
| **SUBCODE: Value of/thoughts around newspaper announcements** | Use when an individual speaks to the utility or lack thereof of traditional newspapers for dissemination of a communications campaign. |
| **SUBCODE: Value of/thoughts around YouTube/Internet-based news channels** | Use when an individual speaks to the utility or lack thereof of YouTube or other Internet-based news channels for dissemination of a communications campaign. |
| **SUBCODE: Value of/thoughts around WhatsApp/WeChat/other communication apps** | Use when an individual speaks to the utility or lack thereof of communication apps like WhatsApp, WeChat or others for dissemination of a communications campaign. |
| **SUBCODE: Information delivery methods for CHWs and providers** | Use when a participant describes effective methods of information and awareness campaign delivery for community health workers, healthcare providers, and other professionals. |
| **SUBCODE: Value of and thoughts around messaging for healthcare provider and hospitals** | Use when a participant offers thoughts on the utility of messaging or information targeted specifically to doctors and hospitals and their utility in disseminating a communications campaign. |
| **SUBCODE: Value of and thoughts around messaging for government** | Use when a participant offers thoughts on the utility of messaging or information targeted specifically to government officials and their utility in disseminating a communications campaign. |
| **SUBCODE: Value of and thoughts around personal testimonials** | Use when a participant discusses there's utility of personal testimonies of people living with or affected by hepatitis B. |
| **SUBCODE: Age/generational differences in obtaining health information** | Use when an individual shares about generational differences in sources through which health information is obtained. |
| **SUBCODE: Age/generational differences in communication preferences** | Use when an individual shares about generational differences in which communication channels are most appropriate for health information campaigns. |
| **SUBCODE: Spoken language resource preferences (English, native language, bilingual)** | Use when an individual indicates appropriate languages in which spoken materials should appear in a communications campaign. |
| **SUBCODE: Written language resource preferences (English, native language, bilingual)** | Use when an individual indicates appropriate languages in which written materials should appear in a communications campaign. |
| **SUBCODE: Age differences in language preferences** | Use when an individual shares about generational differences in language preferences, including preferences for spoken and written languages. |
| **PARENT CODE: Culturally appropriate presentation of awareness campaign** | Use when an individual indicates how best to create a communications campaign that is culturally appropriate. |
| **SUBCODE: Importance of messenger over message** | Use when a participant describes the importance of a person relaying a message over the message itself. |
| **SUBCODE: Words/phrases/images/colors to use** | Use when an individual speaks to the utility of particular words, phrases, images, or colors in a communications campaign. |
| **SUBCODE: Words/phrases/images/colors to avoid** | Use when an individual speaks to the lack of utility of particular words, phrases, images, or colors in a communications campaign. |
| **SUBCODE: Value of use/avoidance in religious messaging or mention of specific religious beliefs** | Use when an individual speaks to the utility or lack thereof of messaging that is religious in nature. |
| **SUBCODE: Value of use/avoidance in cultural messaging or mention of specific cultural beliefs/proverbs** | Use when an individual speaks to the utility or lack thereof of messaging using cultural beliefs or proverbs. |
| **SUBCODE: Value of age-specific messaging** | Use when an individual speaks to the utility or lack thereof of age-specific messaging. |
| **SUBCODE: Value of gender-specific messaging** | Use when an individual speaks to the utility or lack thereof of gender-specific messaging. |

**Supplementary Table 1.** The table above includes the codes and subcodes identified and used during qualitative data analysis in this study.

## Supplementary Tables

**Interpersonal Communication Preferences Thematic Table**

| **Themes** | **Subthemes** | **Quotes** |
| --- | --- | --- |
| **Explaining the Link Between hepatitis B and Liver Cancer** |  | *“Can you explain to me what’s the difference between hepatitis and liver cancer? I don't really know the difference.”* - Chinese-Cantonese participant  *“Well, what caught my attention as to when this disease degenerates and turns into cancer, is because someone might be asymptomatic.”* - Haitian participant |
| **Identified Interpersonal Communication Preferences** | **Messenger:**  **Doctors or trusted community health workers** | *“We encourage doctors to share information because people don’t listen to others but to doctors. Many older people normally debate a lot with others but usually agree with doctors.”* - Vietnamese participant  *“When we work with people from the community, they know who to speak to, and they know what the crowd is comfortable with, and the level of understanding from our community.”* - Micronesian participant  *“... it could be community-based organizations and they usually - people focus on the messenger, not the message. So, if the message is given by somebody that they trust, that they know who speaks their language, who understands their culture, I think it can go a long way.”* - Somali participant |
|  | **Messenger: Faith leaders** | *“If we were to reach out to certain religious groups, I think it's good to touch base with maybe their leaders…to check what message to not give, or things to avoid.”* - Chinese-Mandarin participant |
|  | **Messenger: Gender-specific** | *“There are some things that men only say amongst men, women only say amongst women.”* - Micronesian participant  *“If you're a woman, women should be teaching the class or training. If there's a male, a male should be comfortable.”* - Somali participant |
|  | **Preferred Language** | “*I try to speak English, but then I try to also speak the local dialect that I think most people understand and speak to them about what I'm trying to say or what the important message is. So, I use both.”* - key informant  *“Maybe you want to talk to them in a seminar form, maybe like in the mosque setting, I think it's where you speak Yoruba, then maybe when you're passing like a flyer that you can pass to people English will be more appropriate.”* - Nigerian participant |
|  | **Preferred Setting: Community** | *“You need to hold seminars and invite the Asian communities to attend them. Why? Because general public don't know about hepatitis antibody, cirrhosis and the differences between these diseases.”* - Chinese-Cantonese participant  *“I would really, certainly encourage this information to be given in a place where there's already Hmong people feeling really comfortable. And they know ahead of time that it's going to be there, and again, putting it in an atmosphere that allows for a communal feel.”* - Hmong participant  *“I think churches are very powerful so, if we do it, maybe using the church platform, because they still attract many people.”* - Ethiopian participant  *"Well, I have questions. ‘Who should I talk to?’ I don't know. To me, I just like to speak with people versus just clicking a link.”* - Hmong participant |
|  | **Preferred Setting: Healthcare** | *“I think you need to get hospitals to be involved in this. Otherwise, people won’t pay attention. They will be like, 'Why should I get these shots?’.”* - Chinese-Cantonese participant  *“If the doctors, because I know I go there for one thing, and then they see this. If they can explain to me clearly as to why this is important, or this is necessary to take, I will take it.”* - DC West African  *“Doctors have it in there, not just outside in the waiting area, but when they’re in the room. And it’s even better if a doctor could hand it to* [their patients].” - Nigerian participant  *“It's going to be helpful, but at the same time, it's going to be only for the people that go to the doctor's office -if I don't go to the doctor's office, I won’t know that something like that is there.”* - Nigerian participant |
| **Preferred Messaging Channels** | **Mobile Messaging Applications** | *“The only way that we communicate is through WhatsApp.”* - African and Caribbean Advisory Committee member  *“What I know is everybody, I think a lot of Africans use WhatsApp.”* - key informant  *“There's some people they have access to the social media, and some elders that don't have access to social media beside WhatsApp group and all those stuff, that's where they communicate it.”* - Somali participant  *“I think those are too intrusive.” -* Hmong participant’s thoughts around WhatsApp, WeChat, and other communication apps  *“If they send something like that to me, and it is not my aunty, who spoke to me, I won’t be included today. I would just push it away.” -* Nigerian participant’s thoughts around WhatsApp, WeChat, and other communication apps |
|  | **Personal Testimonials** | *“When I do education, sometimes I talk about my story. I talk about my experience, but then sometimes I also bring stories that other people have shared with me.”* - key informant  *“I think you got to have a like-person like me go out to the community and say, ‘Hey, look at me, I'm like you. I have it, here's why I have it…here's my story…And here's what it leads to’.”* - Hmong participant  *“Nowadays programs like this, they like to include heroes, where these are survivors or people who are currently having the disease, so people who have hepatitis B can show an example of how to live life with hepatitis B and be successful at it.”* - Micronesian participant  *“People, they want to hear the stories, so they might relate to them, and sometimes they can be learning something from that.”* - Somali participant |
| **Communication Themes** | **Hope-based messaging** | *“When someone projects fear, it would make you feel like you can’t do anything about it. But when you use the message of hope, the person would feel that at least, something can be done about it.”* - Nigerian participant  *“There's softer, understandable speech to welcome it. The more aggressive it is, the more discouraging it will be, and people with withdraw. So, it all depends on the manner, how you present the message.”* - Francophone West African participant  *"I don't think that fear is going to help drive grandpas and grandmas to go and get tested."* - Hmong participant |
|  | **Fear-based messaging** | *“It would be better if it included a message about fear and alertness about this disease because many people have heard of hepatitis B but do not know exactly what it is.”* - Korean participant |
|  | **Quality of Life** | *“I think having the fact that you can still get married and have children, that there's still life after being diagnosed with hepatitis B or having liver cancer.”* - Hmong participant  *“It's also important not to just give information, but also reassurance and comfort that whatever the result, things will still continue as usual, and so information is important, but it should be supplemented with reassurance."* - key informant |
|  | **Respect** | *“When we do community outreach in the Chuukese community, it's very disrespectful to talk about the sexual like, just even mentioning, the different parts of man and woman or like anything that only the men say among themselves or the lady say among themselves.”* - Micronesian participant  *“Well, definitely education is a better approach, because you are going to give them a background and a history that they didn't do something dirty to get it, right? Perhaps you draw the attention to it, that it's nothing that they did just that they were born that way, or because of the socio-economic reasons, and based on studies, and we have this percentage or this number, you have facts, something more tangible to tell a person, and they may not have been aware, but letting them know that it's more probable that many people in your community have it, but it's unknown, so they are not alone in this.”* - DC West African participant |
|  | **Community** | *“We have to inform the people around them not to isolate them, on the contrary to help them. Bring consciousness to all the community.”* - Haitian participant  *“People with increased risk for liver cancer, we need to do a lot of again education and other kinds of psychological, social help, or spiritual help even for some folks…they need some sort of support system in order to maintain their healthier lifestyle to reduce their risk as much as possible*.” - Marshallese participant  *“We don't like to do screening or any testing. But if it comes down to this will help your family, then people were more willing and wanted to do it.”* - AAPI Advisory Committee member  *“With almost every African, family is very important to us. So, maybe to draw on that idea that if you take care of yourself, then you will be there to take care of your family.”* - key informant |
|  | **Faith-based Messaging** | *“*[He was] *very encouraging and he had like some quotes from the Quran when he was saying public health is like religious duty and then we all have the responsibility to care for one another.”* - Somali participant  *“As a faithful Muslim everything is there. Islam asks you to take care of your health because you must be healthy to praise. Even the Prophet, when he was sick, He had to go and see the doctor. In the message, you can show that everyone gets sick. Even our Prophet... Go get treated.”* - Francophone West African participant |
|  | **Science-based Messaging** | *“Just be specific about the health issue, nothing about -- this is not about religion issue at all.”* - Nigerian participant  *“I think our information relates to science, medicine, and medical knowledge, it has nothing to do with religions or beliefs, we should talk only about science, we have no reason to mention about religions or beliefs.”* - Vietnamese participant  *“Religion stuff is really touchy. And you really push away a lot of people, prospective people that would really benefit from the information.”* - Hmong participant |

**Supplementary Table 2.** The thematic table presents illustrative quotes for each qualitative theme and the subcategories within each theme.
